# Environmental pollution, apolipoprotein A-1 autoantibodies and cardiovascular risk: evidence from a geospatial cross-sectional study^☆^

**DOI:** 10.1016/j.athplu.2026.100566

**Published:** 2026-05-07

**Authors:** Noé Fellay, Sabrina Pagano, Pedro Marques-Vidal, Peter Vollenweider, Silvia Stringhini, David De Ridder, Julien Vaucher, Aurelien Thomas, David Carballo, Idris Guessous, Mayssam Nehme, Stéphane Joost, Nicolas Vuilleumier

**Affiliations:** a Geospatial Molecular Epidemiology Group (GEOME), Laboratory for Biological Geochemistry (LGB), School of Architecture, Civil and Environmental Engineering (ENAC), Ecole Polytechnique Fédérale de Lausanne (EPFL), Lausanne, Switzerland; b Division of Laboratory Medicine, Diagnostics Department, Geneva University Hospitals, 1205, Geneva, Switzerland; c Department of Medicine Specialities, Medical Faculty, Geneva University, Switzerland; d Department of Medicine, Internal Medicine, Lausanne University Hospital and University of Lausanne, Lausanne, Switzerland; e Department of Health and Community Medicine, Faculty of Medicine, University of Geneva, Geneva, Switzerland; f Division of Primary Care Medicine, Geneva University Hospitals, Geneva, Switzerland; g Group of Geographic Information Research and Analysis in Population Health GIRAPH, Geneva, Switzerland; h Department of Internal Medicine and Specialties, Division of Internal Medicine, Fribourg Hospital and University of Fribourg, Fribourg, Switzerland; i Unit of Forensic Toxicology and Chemistry, CURML, Lausanne and Geneva University Hospitals, Lausanne, Geneva, Switzerland; j Faculty Unit of Toxicology, CURML, Faculty of Biology and Medicine, University of Lausanne, Lausanne, Switzerland; k Division of Cardiology, Department of Medicine, Geneva University Hospitals, Switzerland; l La Source School of Nursing, University of Applied Sciences and Arts Western Switzerland (HES-SO), Lausanne, Switzerland

**Keywords:** Environmental pollution, Cardiovascular diseases, Autoantibodies, Apolipoprotein A-1, Spatial analysis, Life expectancy

## Abstract

**Aims:**

Autoantibodies against apolipoprotein A-1 (AAA1) are independent cardiovascular risk factor in the general population. Genetic factors and viral infections have been linked to AAA1 occurrence. We studied the association between environmental pollution and AAA1 response and their relationship with years of life lost or gained (YPLLG).

**Methods:**

We conducted a cross-sectional study involving 1867 individuals recruited between 2016 and 2018 in Geneva, Switzerland, characterized for serum AAA1 levels. Exploratory and confirmatory geospatial analyses of AAA1, air pollution (NO_2_, PM_2_._5_, PM_10_), proximity to polluted sites, and YPLLG were performed. Associations with AAA1 were adjusted for premature mortality risk factors using Systemic Coronary Risk Evaluation 2 (SCORE2), while associations with YPLLG were adjusted for income, nationality, NO_2_ levels, and proximity to polluted sites.

**Results:**

Significant geographic clustering of high AAA1 serum levels (hotspots) were identified in urban areas with heavy traffic, associated with higher air pollutant levels (median NO_2_ 26.90 μg/m^3^ in hotspots vs. 23.70 μg/m^3^ in non-hotspots, *p* < 0.001), and greater density of polluted sites (median weighted pollution score 66.8 vs. 52.1, *p* = 0.008). AAA1 geographic hotspots corresponded to areas showing a median reduction of four years in population life expectancy, based on mortality records (2009–2016) (*p* < 0.001), even after adjustments.

**Conclusions:**

While causal inference cannot be drawn due to the cross-sectional design, AAA1 hotspots overlapped regions of higher air and soil pollution and reduced life expectancy. Determining causality and whether specific pollutants amplify the AAA1 response will be key to validate these hypothesis-generating findings.

## Highlights

• Geographic hotspots of elevated AAA1 levels were identified in an urban general population.
• AAA1 hotspots overlapped areas of higher air pollution and polluted-site density.
• AAA1 hotspots were associated with reduced population life expectancy.
• Associations remained after adjustment for cardiovascular risk and environmental factors.

## Graphical abstract

**Graphical abstract**: Visual representation of how environmental pollution may contribute to direct and indirect toxicity, potentially leading to the induction of autoantibodies against apolipoprotein A-1, known to be associated with increased cardiovascular disease risk and reduced life expectancy in the general population.

**Figure:**
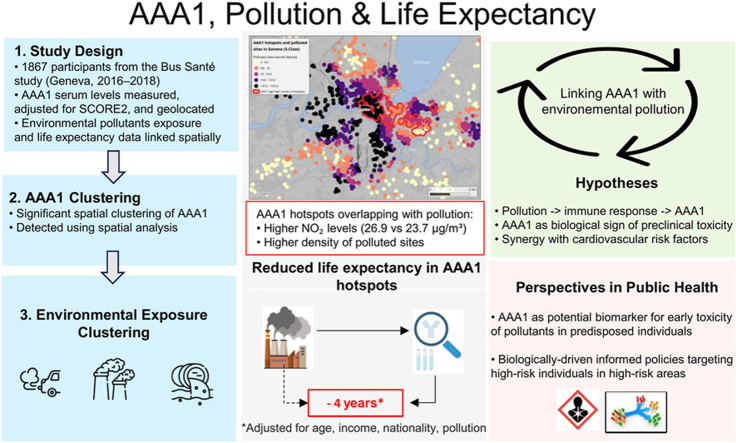

## 1 Introduction

The link between environmental pollutants and adverse health outcomes is an active area of investigation, bridging environmental science, epidemiology, and biology. Several environmental pollutants have been identified as potential contributors to a broad range of pathologies, including cardiovascular, respiratory, liver, cancers and autoimmune diseases [1,2]. Major categories of environmental pollutants include air and noise pollution, persistent organic pollutants, heavy metals, pesticides and plasticizers [3,4].

Health risks may emerge over time due to pollutant bioaccumulation, even at levels below regulatory thresholds [5], leaving pre-symptomatic detection of exposed individuals prone to develop long-term complications and unmet medical and public health needs. Furthermore, certain air pollutants (such as fine-particular PM_2.5_, exposure to traffic related pollution and diesel-exhaust) have been shown to have both short term as well as long term cardiovascular health effects [[6], [7], [8]]. For this purpose, developing risk stratification tools combining geographic pollutants exposure clusters with individual biological data capturing pre-clinical toxicity could prove valuable. The usefulness of such a generic approach has been best exemplified in the context of the COVID-19 pandemic, where geospatial clustering analyses of individual biological data enabled real-time pandemic tracking and informed data-driven public health policies [9].

Stemming from the need for early detection and prevention strategies, biomarkers that can reveal the presence of ongoing pathophysiological processes before clinical symptoms appear are crucial. Preceding overt disease for many years [10] and fulfilling standard biomarkers’ requirements, autoantibodies could represent appealing biomarkers for such purposes. Among them, autoantibodies against apolipoprotein A-1 (AAA1) – the major protein fraction of high-density lipoprotein particles – are of particular interest in the context of cardiovascular (CV) diseases. AAA1 have been shown to represent an independent cardiovascular risk factor with a documented propensity to predict adverse cardiovascular outcomes in several study populations and also shown to promote atherogenesis in mice and *in vitro* (reviewed in Ref. [11]). However, the reasons for the relatively high prevalence of AAA1 seropositivity in the general population (approximately 20-30%) remain poorly understood. So far, several determinants of elevated AAA1 have been identified, including genetic predisposition, previous myocardial infarction, certain environmental factors, such as mRNA virus infections (human immuno-deficiency virus, hepatitis C virus, and SARS-CoV-2) and niacin therapy reviewed in Refs. [11,12]. However, these factors do not fully explain the high seropositivity prevalence in the general population. The mechanisms by which pollutants drive pathogenic autoantibody formation are poorly understood but likely involve loss of self-tolerance (immunotoxicity), neo-epitopes formation (oxidative stress, tissue damage), host genetic/epigenetic susceptibilities, and epitope spreading [[13], [14], [15], [16]].

Although not yet addressed for AAA1, preliminary and unpublished results from our group indicate that AAA1 hotspots coincide with polluted areas in another Swiss region and that cadmium exposure in mice induces an AAA1 response (data not shown).

Therefore, we explored the associations between air pollution (NO_2_, PM_2_._5_, and PM_10_), referenced polluted sites, 10-year CV risk, drinking water distribution supply and individual AAA1 circulating levels in participants living in the state of Geneva (Switzerland) and recruited in the *Bus Santé* study. Finally, we assessed the impact of these environmental factors and AAA1 on years of life lost or gained (YPLLG) compared to life expectancy at birth.

## 2 Methods

### 2.1 Study population

#### 2.1.1 Bus Santé

The study consists of 1987 individuals (aged 18-75 years) enrolled in *Bus Santé* cohort in Geneva between February 2016 and January 2018. Participants were randomly sampled from the register of the Geneva population office. There were no specific exclusion criteria besides the inability to provide informed consent or complete the study procedures.

The study was approved by the Geneva Ethics Council for Research (Protocol Nr: CCER16-363), and written informed consent was obtained by patients prior to enrolment in accordance with the Declaration of Helsinki.

For our main analysis, only 1867 individuals were included as 115 of them had missing values for SCORE2 computation and 5 of them resided outside Geneva. The comparison between included and excluded individuals is reported in the Supplementary Results (3.1). Baseline demographic and clinical characteristics are presented in Table 1.

**Table 1:** **Baseline individual characteristics and participant-assigned environmental exposures (N = 1867).** Values are median [IQR] or n (%). OD: optical density, IQR: interquartile range, HDL: high-density lipoprotein, SBP: systolic blood pressure. NO_2_/PMs from PolluMap (2015); polluted-site densities from cantonal inventories (kernel density, arbitrary units). Night noise reported as dB for road and rail. Land surface temperature in °C. AAA1 analyses were adjusted only for SCORE2. YPLLG and related contextual covariates (income, nationality) originate from an independent mortality dataset (Ladoy et al., 2021) and were used only in spatial analyses described in the Supplementary Material §2.3.4

	Median [IQR]	N (%)	95% CI
**Demographics**			
Age (years)	45.6 [35.8-56.5]		
Sex (Female)		981 (52.5%)	[50.3–54.8]
**Health Metrics**			
AAA1 (OD)	0.46 [0.31-0.65]		
Adjusted AAA1 (OD)	0.39 [0.24-0.59]		
AAA1 positivity (% positive)		502 (26.9 %)	[24.9–28.9]
SCORE2 (%)	1.6 [0.7-3.8]		
Total Cholesterol (mmol/L)	5.20 [4.50-5.90]		
HDL Cholesterol (mmol/L)	1.56 [1.27-1.90]		
SBP (mm Hg)	116 [107-128]		
Diabetes (% yes)		107 (5.7%)	[4.8–6.9]
Previous myocardial Infarction (%)		26 (1.4%)	[0.9–1.9]
Angina Pectoris (%)		21 (1.1%)	[0.6–1.6]
**Environmental exposures (assigned to each participant)**			
NO_2_ (μg/m^3^)	24.1 [21.6–26.6]		
PM_10_ (μg/m^3^)	16.4 [15.6–17.1]		
PM_2.5_ (μg/m^3^)	11.1 [10.7–11.6]		
Polluted-site density (unweighted)	48.1 [11.7–101.9]		
Polluted-site density (weighted)	52.4 [11.9–122.6]		
Road night noise (dB)	43.5 [38.0–49.0]		
Rail night noise (dB)	0.0 [0.0–10.0]		
Land surface temperature (°C)	24.1 [22.9–25.2]		
**Behaviours and Conditions**			
Smoking (% smokers)		398 (21.3%)	[19.5–23.2]
Insomnia (% yes)		715 (38.3%)	[36.1–40.5]
Previous hospitalizations (%)		176 (9.4%)	[8.1–10.7]

#### 2.1.2 Samples processing and AAA1 levels assessment

Serum AAA1 levels were measured according to a validated method (reviewed in Ref. [11]) as explained in the supplemental material.

### 2.2 Study objectives

The first objective was to determine the existence of significant AAA1 geographic clusters in Geneva.

The second objective was to explore the associations between these AAA1 clusters with i) air pollution (NO_2_, PM_2_._5_, PM_10_) and ii) referenced polluted sites in the state of Geneva.

The third objective was to establish the possible association between AAA1 clusters and years of life lost or gained (YPLLG). YPLLG was calculated as the difference between individual ages at death and the expected life expectancy at birth, using data from individual death records (n = 22751) from official records between 2009 and 2016 [17].

Finally, sensitivity analyses considering additional environmental factors such as water pollution (drinking water supply distribution), noise pollution, and land surface temperature were carried out to challenge the robustness of our study's conclusions and to account for potential confounding variables. Detailed information is available in supplemental section 2.4.

### 2.3 State data collection

#### 2.3.1 Air pollution data collection

Air pollution data (NO_2_, PM_2_._5_, PM_10_) for the year 2015 were obtained as explained in the supplemental material 2.3.2.

#### 2.3.2 Identification of polluted sites in the state of Geneva

Data on polluted sites was obtained as explained in the supplemental material 2.3.3.

#### 2.3.3 Assessment of the difference in life expectancy at birth

YPLLG data were sourced from a previous study on geographical inequalities in life expectancy in Geneva, Switzerland [17]. YPLLG is a validated metric defined as the difference in years between the age at death and the individual's life expectancy at birth [17]. Positive YPLLG values indicate years gained, while negative YPLLG values represent years lost. YPLLG results were derived at an individual level from a specific registry devoid of adequate information to generate SCORE2 (17).

### 2.4 Sensitivity analyses and contextual environmental data

Sensitivity analyses and related details are presented in supplemental material section 2.4.

### 2.5 SCORE2 computation

SCORE2 and SCORE2-OP (hereinafter, SCORE2) risk scores, were respectively computed as detailed in the supplemental material to cover ages up to 89 years-old [18,19].

### 2.6 Statistics

Results were reported as proportions or medians when appropriate and provided with corresponding 95% confidence intervals (95%CI) and/or interquartile ranges (IQR), respectively. Given the exploratory nature of this study, the current study number was determined by complete data set availability only, without prior power calculation.

The spatial distributions of serum AAA1 levels, air (NO_2_, PM_2_._5_, and PM_10_), and soil pollution were analysed using local indicators of spatial association (LISA), specifically the local Moran's I statistics, a standard method to identify areas where neighbouring values are significantly higher or lower than expected by chance [20]. Significance was assessed using 999 random permutations, and clusters were considered significant with α ≤ 0.05. After sensitivity analysis using bandwidths of 800, 1000, 1200 and 1400 m, a spatial lag of 1000 m was selected consistent with previous studies in Geneva [21,22]. To preserve anonymity in spatial visualizations, all point coordinates were geomasked within a variable-radius zones (≥25 m up to 400m), scaled to local population density (more detailed information is available in supplement Section 2.3.1). Five cluster types were identified: high-high (hotspots), where neighbours with high AAA1 levels surround individuals with high AAA1 levels; low-low (cold spots), where low values cluster together; high-low, low-high, and a neutral class, (no spatial dependence). Our primary analyses focused on hotspots and cold spots compared to the neutral geographic space to streamline data interpretation, using a 50m buffer zone. AAA1 cluster analyses were adjusted for SCORE2. The Mann-Whitney *U* test was used to compare AAA1 levels in the hotspots versus cold spots. Further detailed information is available in the Supplementary Material (2.3.1) and in the result section of the online supplement.

For YPLLG, a buffer zone of 100 m was considered, being a common trade-off value between precision and statistical stability. This buffer assumed that individuals residing near hotspots of AAA1 would exhibit similar YPLLG values. YPLLG analyses were adjusted for median age, income, nationality, NO_2_ exposure, and proximity to polluted sites, according to the median regression methodology described in Ladoy et al. [17] and detailed in the online supplement (Section 2.3.4). Environmental and YPLLG differences across AAA1 clusters were tested using Kruskal-Wallis and Mann-Whitney U tests. Pearson and Spearman correlations assessed linear associations between YPLLG and environmental variables.

Finally, Geographically Weighted Regressions (GWR) between AAA1 and the variables (NO_2_, PM_10_, PM_2.5_, night traffic noise, night train noise, land surface temperature, and polluted sites (with and without weighting)) were conducted for further analysis. More information is available in the online supplement (Section 2.6). For all analyses, a threshold of α ≤ 0.05 was used to identify statistically significant results. Maps were created to illustrate the different co-occurrences of clusters of interest using QGIS 3.40, statistical analyses were performed using Python 3.8.

## 3 Results

### 3.1 Baseline demographic characteristics

Of the 1987 participants, 1867 were included in the main analysis (minimal bias from exclusions; Supplementary Results 3.1). Baseline demographic characteristics of the 1867 study participants are summarized in Table 1. Briefly, 52.5% were female and 47.5% were male. Participants had a median age of 45.6 years [IQR: 35.8–56.5]. The median AAA1 level was 0.46 OD units [IQR: 0.31–0.65], and after adjustment for SCORE2, the median AAA1 level was 0.39 OD units [IQR: 0.24–0.59]. AAA1 seropositivity was 26.9% (95% CI 24.9-28.9). Participants were overall healthy with low SCORE2 risk (median 1.6%, IQR: 0.7–3.8) and few cardiovascular events (≤1.5%) (Table 1).

#### 3.1.1 Assessment of AAA1 hotspots in the state of Geneva

**Fig. 1:**
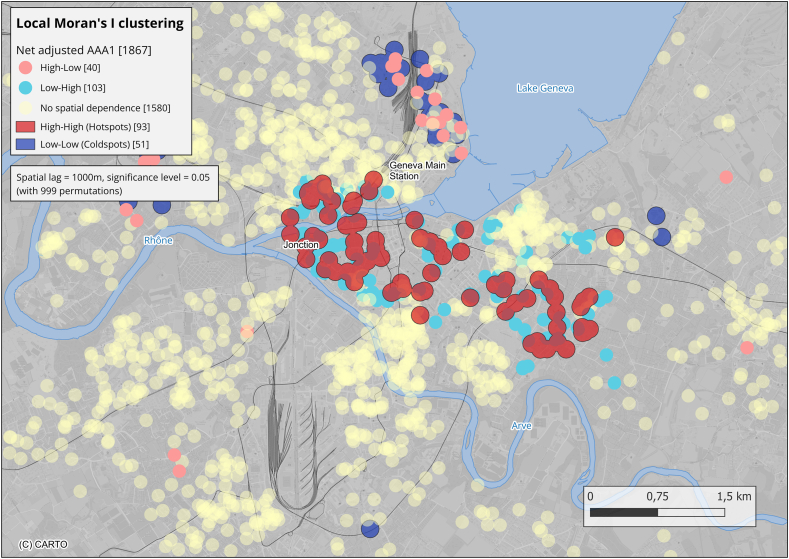
Zoom displaying Local Moran's I of adjusted AAA1 results (2016-2018, ages 20-75 years) for the city of Geneva. The spatial distance was set to 1000 m, with a significance level of 0.05 and 999 permutations. Red buffers indicate high-high clusters (hotspots; high values surrounded by high mean values, n = 93), blue buffers indicate low-low clusters (cold spots; low values surrounded by low mean values, n = 51), teal dots indicate low-high clusters (low values surrounded by high mean values, n = 103), and pink dots indicate high-low clusters (high values surrounded by low mean values, n = 40). For neutral areas (no spatial dependence), n = 1580. For the full extent of the figure, refer to Supplementary Fig. 2.

Fig. 1 shows significant AAA1 hotspots, mainly in dense urban areas near railway lines and motorways in Geneva, with significantly higher median AAA1 levels than cold spots (0.69 OD [IQR: 0.61–0.92] vs. 0.40 OD [IQR: 0.28–0.44], p < 0.001). This pattern held across bandwidths of 800-1400 m (Supplementary Fig. 1), confirming cluster robustness. The median AAA1 level for the neutral zones (no spatial dependence) was 0.45 OD (IQR: 0.31-0.65). Supplementary Fig. 2 displays these clusters with a spatial bandwidth of 1000 m.

#### 3.1.2 AAA1 hotspots and air pollution

**Fig. 2:**
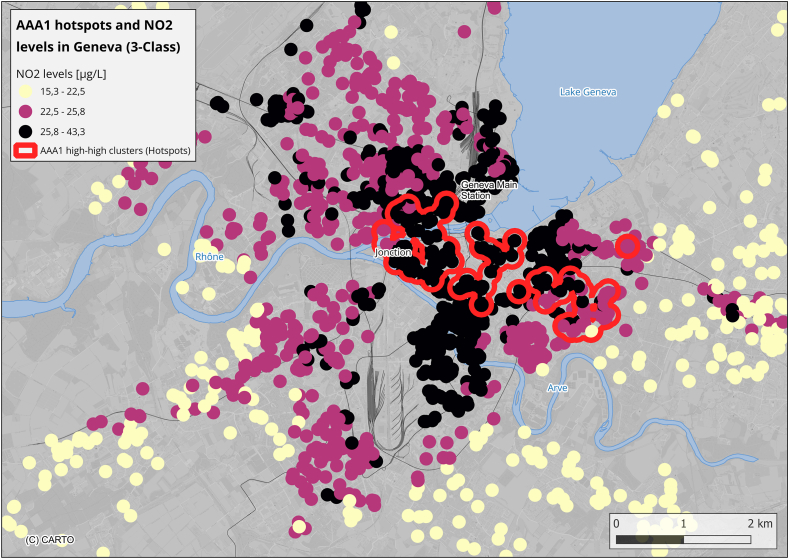
AAA1 high-high clusters (hotspots) are shown in red, overlaid with NO_2_ levels categorized into three equal quantile classes: first quantile (white), second quantile (light blue), and third quantile (dark blue). A 150-m buffer around hotspots was created for visualization.

AAA1 hotspots overlapped substantially with areas of elevated air pollution (Fig. 2, Supplementary Figs. 3–4). The Kruskal-Wallis test revealed significant differences in NO_2_ levels across clusters (p < 0.01). Median NO_2_ in hotspots was 26.9 μg/m^3^ [IQR: 24.4–30.2], versus 23.7 μg/m^3^ [IQR: 21.4–26.2] in neutral and 25.7 μg/m^3^ [IQR: 22.2–26.5] in cold spots (p < 0.01). Similar trends were observed for PM_2_._5_ and PM_10_ (p < 0.01 for both; Supplementary Table 1).

#### 3.1.3 AAA1 hotspots and polluted sites

**Fig. 3:**
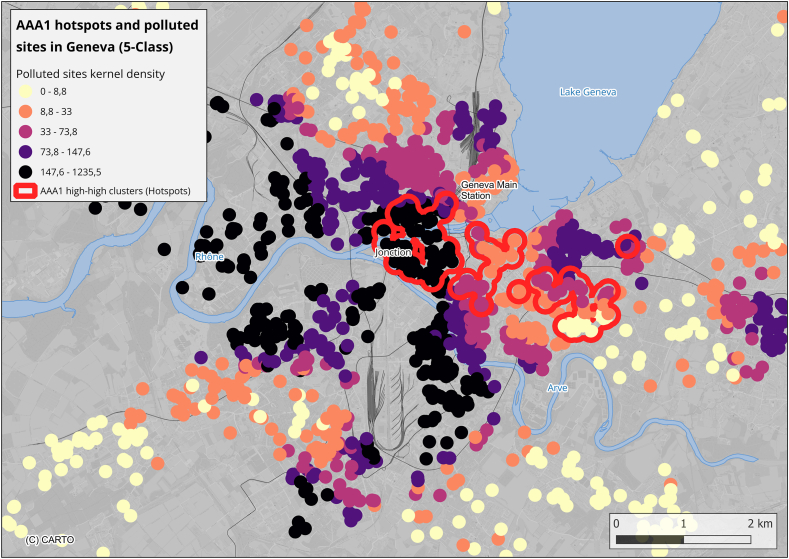
AAA1 high-high clusters (hotspots) are shown in red, overlaid with the density of polluted sites categorized into five equal quantile classes. A 150-m buffer around hotspots was created for visualization.

Fig. 3 shows a notable spatial overlap between AAA1 hotspots and polluted sites density in Geneva. AAA1 hotspots had a median pollution score of 66.8 [IQR: 13.7–273.6], compared to 52.1 [IQR: 11.5–113.2] in the neutral class (p = 0.008, Mann-Whitney *U* test). Differences were close to significance in the unweighted model (p = 0.07) but became significant in the weighted model (p = 0.004), suggesting that AAA1 clustering corresponds more closely to zones with higher intensity of soil pollution, rather than merely to the number of sites.

#### 3.1.4 AAA1 hotspots and years of life lost or gained

**Fig. 4:**
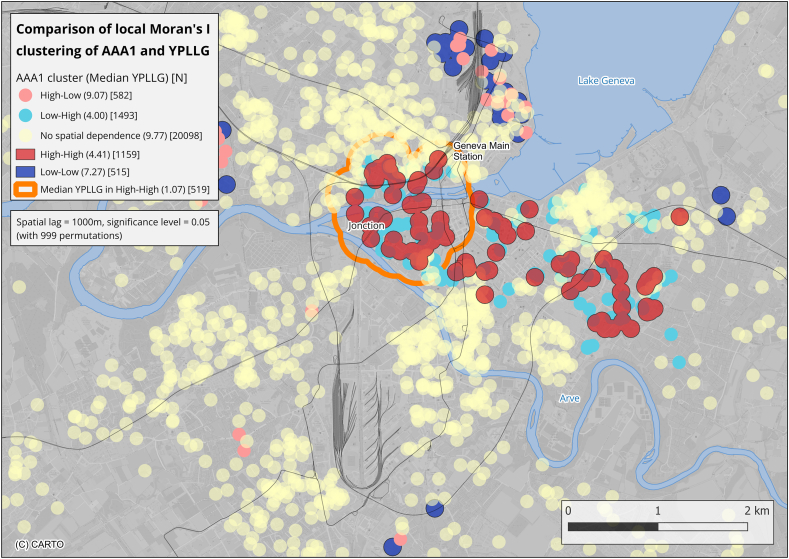
Median YPLLG values within AAA1 cluster buffers (100m): high-high (hotspots), low-low (cold spots), high-low, low-high, and neutral zones (no spatial dependence). The area highlighted with an orange polygon indicates a region with significantly lower median YPLLG values. The “n" displayed on the figure represents the number of YPLLG values from the dataset that fall within each respective AAA1 cluster. The spatial distance to find AAA1 clusters was set to 1000 m, with a significance level of 0.05 and 999 permutations. Red buffers indicate high-high clusters (hotspots) (e.g., high values surrounded by high mean values, n = 93), blue buffers indicate low-low clusters (low values surrounded by low mean values, n = 51), teal dots indicate low-high clusters (low values surrounded by high mean values, n = 103), and pink dots indicate high-low clusters (high values surrounded by low mean values, n = 40).

To explore the link between AAA1 levels and life expectancy, AAA1 hotspots were compared with YPLLG values using a 100-m buffer. The orange area in Fig. 4 highlights a zone where people were shown to lose more years of life [17]. This area partially overlaps with an AAA1 hotspot in the centre of Geneva near major railways and motorways. The median YPLLG in AAA1 hotspots was 4.41 years [IQR: −13.33 to 14.61], significantly lower than in cold spots (7.27 years [IQR: −10.97 to 18.61], p = 0.002) and the neutral class (9.77 years [IQR: −3.72 to 19.29], p < 0.001).

Table 2 shows that AAA1 hotspots systematically lost more years of life than those in the cold spots or the neutral areas, even after full adjustment in Model 3 (median age, income, nationality, NO_2_, and polluted site density).

**Table 2:** **Median and IQR YPLLG values and statistical comparisons for different AAA1 clusters within a 100m buffer distance.** The neutral class corresponds to the class where no spatial dependence was found. Model 1, unadjusted. Model 2, YPLLG residuals adjusted for median age, income, and nationality (median regression). Model 3, YPLLG residuals adjusted for median age, income, nationality, NO_2_ levels, and polluted sites where applicable. Negative residuals = fewer years than expected; positive = more years than expected. One can see that people in high-high AAA1 clusters lose approximately 4 years of life when compared to low-low AAA1 clusters or the neutral areas.

Model	High-High clusters (Hotspots) (n = 1159)	Low-Low clusters (Cold spots) (n = 515)	Neutral areas (n = 20098)	Kruskal-Wallis p-value	Mann-Whitney p-value		
					HH vs. LL	HH vs. Neutral	LL vs. Neutral
1	4.41 [-13.33; 14.61]	7.27 [-10.97; 18.61]	9.77 [-3.72; 19.29]	<0.001	0.002	<0.001	0.003
2	−4.30 [-21.17; 6.81]	0.23 [-17.68; 11.69]	0.33 [-12.48; 9.88]	<0.001	<0.001	<0.001	0.910
3	−3.12 [-20.28; 7.83]	0.78 [-17.47; 12.64]	0.24 [-12.39; 9.68]	<0.001	<0.001	<0.001	0.417

Across all buffer sizes (50-200m), YPLLG differences between AAA1-hotspot and non-hotspot locations were directionally consistent with gradual attenuation at larger distances (Supplementary Tables 3–4).

### 3.2 Association between pollutants and years of life lost or gained

We next examined whether reduced life expectancy was directly related to environmental pollution. Supplementary Table 5 shows that regions with greater years of life lost were consistently more polluted across all indicators.

Specifically, individuals in low-low YPLLG clusters (i.e. shorter life expectancy) (n = 1368) had higher median NO_2_ levels of 27.45 μg/m^3^ [IQR: 26.60–29.20], compared to those in the high-high YPLLG clusters (longer life expectancy) (n = 2732), who had median NO_2_ levels of 22.95 μg/m^3^ [IQR: 21.60–24.80] (p < 0.001). Other points (n = 18651) had median NO_2_ levels of 24.20 μg/m^3^ [IQR: 22.10–26.60]. Similar contrasts were observed for PM_10_ and PM_2_._5_.

A Pearson correlation analysis revealed weak but significant negative correlations between YPLLG and NO_2_ levels (r = −0.076, p < 0.001), PM_10_ levels (r = −0.082, p < 0.001), and PM_2_._5_ levels (r = −0.083, p < 0.001). These results were replicated when using Spearman's correlation. Noise showed weaker or inconsistent trends, though low-low YPLLG areas were generally noisier than high-high areas (Supplementary Table 5).

### 3.3 GWR and ordinary linear regression

Compared with the global regression, the GWR model provided slightly better overall explanatory power (Supplementary Table 7), suggesting spatial variability in the associations between AAA1 levels and environmental factors. Localised positive and negative associations appeared across Geneva (Supplementary Fig. 5a–h; Supplementary Table 7).

Only a limited number of sites reached statistical significance: for example, for NO_2_, 36 positive and 6 negative t-values (p ≤ 0.05) were detected among 1602 locations, with a global beta1 coefficient of 0.054 (p = 0.033).

In the global model, PM_2_._5_ and PM_10_ were also significantly associated with AAA1 levels, with a beta1 coefficient of 0.059 (p = 0.020) and 0.053 (p = 0.036), respectively.

For YPLLG, most significant t-values were negative (79/1569 locations), consistent with lower life expectancy in areas with higher AAA1; however, positive associations also occurred sometimes, and none were concentrated within the AAA1 hotspots (Supplementary Fig. 5h).

### 3.4 Sensitivity analyses

#### 3.4.1 Water pollution

Supplementary Fig. 2 shows that in less urbanized areas, AAA1 hotspots were predominantly located within one of the four regions served by the Arve River known to be chronically polluted by heavy metals, and by Cadmium in particular [23].

Supplementary Table 8 shows that Region 4 had higher nitrates (∼4.4 mg/L) and greater hardness, calcium, and magnesium than the regions supplied by other water sources.

#### 3.4.2 Noise pollution

Night-time road-traffic noise varied significantly between clusters (Supplementary Table 9). AAA1 hotspots (47.0 dB [IQR: 42.0–55.0]) were noisier than both cold spots (45.0 dB [IQR: 40.0–48.0], p = 0.001) and neutral areas (43.0 dB [IQR: 37.0–48.0], p < 0.001).

Night train noise showed a similar though less pronounced pattern. Overall, AAA1 clusters exhibit higher nocturnal noise than neutral areas.

#### 3.4.3 Land surface temperature

Land surface temperature differed slightly among clusters (p < 0.001) but did not significantly distinguish AAA1 hotspots (24.1 °C [IQR: 22.5–25.3]) from neutral areas (24.00 °C [IQR: 22.83–25.12], p = 0.622) (Supplementary Table 10).

## 4 Discussion

The primary finding of this study is the non-random spatial distribution of AAA1 levels in the general population of Geneva, with distinct hotspots associated with different forms of environmental pollution, including air, soil, and water contamination. To our knowledge this is the first study to report an association between AAA1 levels and environmental pollution in humans, reinforcing previously established links between pollution and autoimmunity [24,25]. These hotspots align with two major pollution sources: heavy traffic/rail road areas linked to air pollutants and heavy metals emissions, and a specific region sourcing tap water from the Arve River (region 4), known for chronic heavy metals contamination, particularly cadmium [23]. This suggests that heavy metals, and possibly cadmium, may underlie the observed AAA1 patterns. However, this hypothesis remains speculative and requires confirmation in longitudinal studies. Indeed, the absence of strong linear associations between AAA1 and measured pollutants (except NO_2_, PM_10_, and PM_2_._5_) supports this hypothesis suggesting possible involvements of other unmeasured environmental factors. The heterogeneity observed in GWR analyses indicates that local contextual factors, possibly including genetics, lifestyle, synergistic pollutant interactions, or any combination thereof could influence the impact of pollution on AAA1 expression. Because both cadmium and AAA1 are independent CV risk factors associated with poorer outcomes [5,11], further studies are needed to clarify whether heavy metals act as common mediators of pollution-related autoimmunity [24,25], particularly in relation to the AAA1 response.

Second, this study proposes novel insights into the elevated AAA1 seroprevalence in the general population, previously attributed to factors such as myocardial infarction, viral infections, obesity, and niacin therapy [11]. Moreover, it could also contribute to a better understanding of the important international seroprevalences variability reported for various autoantibodies [26].

Our third and key finding is the association between AAA1 hotspots and areas with significant reductions in life expectancy, with individuals in these clusters surviving four years less on average, independently of socioeconomic conditions, NO_2_ levels, and proximity to polluted sites. This difference represents an ecological area-level contrast rather than an individual life-expectancy estimate. Indeed, the mortality and AAA1 datasets consist of different individuals but cover the same geographic regions; the association therefore reflects spatial co-location between high AAA1 areas and regions of shorter life expectancy, not a direct link at the individual level. As a comparison, average life-expectancy losses attributable to ambient air pollution are estimated at ∼1.8-1.9 years worldwide [27] and ∼2.9 years globally across all sources, with European averages generally <1 year [28]. Notably, life expectancy reductions were even more pronounced in the Jonction area, where decreases reached approximately eight years. Our value therefore lies toward the upper range observed in urban European settings and likely reflects localized contrasts in cumulative exposure. While GWR analyses revealed predominantly negative associations between YPLLG and AAA1, their spatial variability suggests additional, localized influences beyond AAA1 and pollution, which was expected. Because all pollution indicators available in this study were significantly associated with lower life expectancy (except for night traffic noise) and because of the established links between environmental pollutants, AAA1, and decreased survival [1,4,[29], [30], [31], [32]], demonstrating that the link between AAA1 and YPLLG was independent of environmental pollution opens innovative perspectives in the field of environmental toxicology. However, the possibility of reverse causality cannot be excluded, as individuals with poorer health or lower socioeconomic status may be more likely to reside in polluted areas.

Current challenges reside in the early identification of individuals at risk when exposed to environmental pollutants because chronic toxic manifestations frequently happen below regulatory individual and environmental thresholds and only after several years of exposure due to bioaccumulation over time, especially for heavy metals [5,33,34]. Because chronic exposure to low levels of environmental contaminants is widespread and unavoidable, an early preclinical toxicity biomarker would be an important first step for developing risk stratification tools and guide targeted eviction policies to mitigate pollution exposure risks. By preceding the onset of clinically overt diseases by many years [11] and showing favourable analytical characteristics, autoantibodies, including AAA1, could represent valuable candidates for such purposes. Future studies are needed to validate or refute such hypothesis.

On a broader level, these results emphasize the potential of integrating georeferenced biological data with exposome information to develop personalized risk stratification tools (see e.g. Ref. [35]). Whether such strategies have the potential to provide personalized, actionable, and data-driven strategies for early intervention and prevention of pollution-related health outcomes remains to be formally demonstrated before any recommendations can be made.

Several limitations warrant consideration. First, the cross-sectional design precludes causal inferences or temporal assessments of the AAA1-pollution and life expectancy associations. Longitudinal studies are necessary to confirm these findings. Furthermore, while the variance analysis in LISA confirmed higher pollution levels in AAA1 hotspots, GWR analyses showed mixed results. This suggests that the relationship between AAA1 and environmental pollution is not uniformly linear and is likely to involve other unmeasured variables. Further work considering other factors including genetic predispositions, other lifestyle factors, and other pollutants such as heavy metals is required to hopefully pinpoint the causes of such geospatial-dependent pattern variability. Although sex was considered through SCORE2 adjustment, stratified analyses by sex were not conducted because the primary aim was to assess environmental exposure effects on AAA1 levels in the general population. Although YPLLG values were adjusted for area-level income, age structure, nationality, and pollution, residual confounders particularly related to traditional CV risk factors, or healthcare access cannot be excluded. Moreover, the lack of individual-level exposure data and reliance on regional environmental measures may have introduced exposure misclassification, as personal behaviours; workplace exposures and residential histories were not accounted for. In addition, pollution data from 2015 slightly preceded the 2016-2018 sampling period. Although spatial pollution patterns remained stable during this time interval in this region [36], small temporal mismatches cannot be formally excluded. The absence of heavy metals data, including cadmium levels, further limits our ability to directly link pollution sources to AAA1 hotspots. Although the GWR analyses highlighted spatial heterogeneity, they could not account for the underlying biological mechanisms or potential interactions between pollutants. Lastly, our findings are based on data from the state of Geneva and do not consider other possible autoantibodies of clinical relevance due to our study protocol. Further studies are required to replicate and generalize these results to populations in different environmental and/or demographic contexts. Because our analyses were conducted in a relatively low-to-moderate pollution context typical of a high-income urban area, our results may not apply to regions with substantially higher pollution burdens, where exposure-response dynamics and sociodemographic factors may differ.

In conclusion, this general population study is the first to report significant geographic clusters of AAA1 hotspots in proximity to polluted areas and heavy traffic density. These AAA1 clusters were associated with reduced life expectancy independently of age, income, air and soil pollution, providing indirect preliminary evidence for AAA1 as a possible biomarker of preclinical toxicity of environmental pollution exposure. Due to the intrinsic limitations of our study design, additional longitudinal and experimental studies are requested to validate the results of this hypothesis-generating study and to demonstrate causality before considering the potential development of public health recommendations based on such biological autoimmune signature.

## Author contributions

**Noé Fellay**: Conceptualization, Data curation, Formal analysis, Investigation, Software, Visualization, Writing – original draft.

**Stéphane Joost**: Conceptualization, Methodology, Supervision, Writing – review & editing.

**Nicolas Vuilleumier**: Conceptualization, Methodology, Supervision, Writing – review & editing.

**Sabrina Pagano**, **Pedro Marques-Vidal**, **Peter Vollenweider**, **Silvia Stringhini**, **David De Ridder**, **Julien Vaucher**, **Aurelien Thomas**, **David Carballo**, **Idris Guessous**, **Mayssam Nehme**: Writing – review & editing.

## Declaration of generative artificial intelligence (AI) in scientific writing

During the preparation of this work, the author used ChatGPT (OpenAI) in order to enhance the clarity and fluency of the English language. After using these tools, the author reviewed and edited the content as needed and takes full responsibility for the content of the publication.

## Funding

This work was supported by the Swiss National Science Foundation (grant Nr 320030-231692 to NV).

## Declaration of competing interest

The authors declare that they have no known competing financial interests or personal relationships that could have appeared to influence the work reported in this paper.
