# Supplementary material for: Environmental pollution, apolipoprotein A-1 autoantibodies and cardiovascular risk: evidence from a geospatial cross-sectional study
Source: Atheroscler Plus. 2026 May 7;65:100566. doi: 10.1016/j.athplu.2026.100566 (PMC13195344; doi:10.1016/j.athplu.2026.100566)
Supplement: Multimedia component 1 [file mmc1.docx]

**Supplementary Material**

**Methods**

2.1.2 Samples processing and AAA1 levels assessment

Blood samples were obtained at the time of outpatient recruitment, and serum was generated from whole blood via centrifugation. Aliquots were then frozen at −80 ˚C until analysed. Serum levels of AAA1 were measured using previously described methods, with the standard AAA1 seropositivity cut-off consisting of AAA1 values above 0.64 optical density measured at 405 nm (reviewed in (1)). This threshold corresponds to the 97.5^th^ percentile of a normal distribution derived from a healthy blood donor population (reviewed in (1)).

2.3.1 Identification of AAA1 geospatial clusters

Five cluster types were identified based on the Local Moran’s I (LISA) analysis:

- High–High (hotspots): individuals with high AAA1 levels surrounded by neighbours with similarly high values.
- Low–Low (cold spots): individuals with low AAA1 levels surrounded by neighbours with low values.
- High–Low: individuals with high AAA1 levels surrounded by neighbours with low values (local outliers).
- Low–High: individuals with low AAA1 levels surrounded by neighbours with high values (local outliers).
- No spatial dependence: individuals showing no significant spatial autocorrelation

**Geomasking procedure**

To preserve participant anonymity in all spatial visualizations, individual point coordinates were geomasked using a fixed-radius donut randomization algorithm implemented in Python. Each point was displaced within an annulus whose minimum and maxmimum radii depended on local population density.

- Population > 100 → 25–75 m
- 50 < Population ≤ 100 → 50–150 m
- 30 < Population ≤ 50 → 75–200 m
- 10 < Population ≤ 30 → 100–300 m

Random angular and radial offsets were drawn from uniform distributions, and points falling outside the Geneva boundary were resampled until they remained within the study area. Geomasking was applied only for visualization purposes and did not affect any statistical analyses or results.

##### **2.3.2 Air pollution data collection**

Air pollution data (NO₂, PM₂.₅, PM₁₀) were obtained from Meteotest ([www.meteotest.ch](http://www.meteotest.ch:)) using the PolluMap dispersion model. The PolluMap model considers both local and imported sources of pollution; for NO₂, total nitrogen oxides (NOx) are first quantified and subsequently converted into NO₂. Detailed information on the PolluMap model and its underlying assumptions is provided in the INFRAS (2020) technical report (2).

Pollution estimates were generated for the year 2015 at a spatial resolution of 20 meters. Although AAA1 samples were collected between 2016 and 2018, 2015 pollution data were used because Swiss air-quality reports indicate that spatial pollution patterns in Geneva have remained stable over recent years, with persistent hotspots along major traffic axes (3).

For each participant’s residential address, a 50-meter buffer was applied, and the median pollutant concentration within this buffer was calculated and assigned to the participant. Other pollutants, such as ozone or volatile organic compounds, were not included because corresponding high-resolution data were unavailable.

**2.3.3 Identification of polluted sites in Geneva County**

Data on polluted sites were obtained from the SITG (“Système d'Information du Territoire à Genève”) website ([www.sitg.ch](http://www.sitg.ch)), established under the Swiss Contaminated Sites Ordinance (CSO, 1998; [www.fedlex.admin.ch/](http://www.fedlex.admin.ch/)), which requires Swiss cantons to maintain an official registry of polluted sites. This inventory includes sites with confirmed or suspected pollution, encompassing facilities or operations either decommissioned or still active where hazardous substances have been used or stored. It also includes permanent storage sites (e.g. landfills and dumps) as well as sites polluted due to extraordinary events, such as operational failures. These data are integrated into the registry of public-law restrictions on land ownership (RDPPF) in the domain of polluted sites (4).

To identify the spatial density of polluted sites, raster heat maps were created, a method commonly used in geographical information systems (GIS) (5) to estimate the density of contaminated areas within urban environments (6).

Each polluted site was assigned a weight based on its contamination level. Using QGIS software version 3.28 (Firenze) and a bandwidth of 1000 meters, a kernel density estimation was performed to generate a continuous surface representing pollution intensity across the region. For each participant, the median density of polluted sites within a 50-meter buffer around their home was calculated to estimate their exposure.

For the main analysis, two different heat maps were generated: one that included weighted polluted sites and another that did not. The detailed process of assigning weights and creating these heat maps is described below.

Polygons representing polluted sites were obtained from official records. Each polygon had an attribute “type_site” indicating its pollution status. Each polygon was assigned an area value based on its geometry, ensuring that larger and more heavily polluted sites received higher weights. The pollution status was categorized and scored as follows:

- *Pollué, pas d'atteinte nuisible ou incommodante à attendre* (Polluted, no harmful or inconvenient impact expected) : 1
- *Pollué, ne nécessite ni surveillance ni assainissement* (Polluted, neither monitoring nor remediation required): 1
- *Pollué, nécessite un assainissement* (Polluted, requires remediation): 4
- *Pollué, investigation necessaire* (Polluted, investigation required): 2
- *Pollué, nécessite une surveillance* (Polluted, requires monitoring): 3
- *Site de stockage, non pollué* (Storage site, not polluted): 1
- *Aire d'exploitation, non pollué* (Operational area, not polluted): 1
- *Lieu d'accident, non pollué* (Accident site, not polluted): 1

A weight of 4 was therefore given to sites that are polluted and require remediation, a weight of 3 to polluted sites that require monitoring, a weight of 2 to polluted sites that must be investigated and a weight of 1 for other sites. The bigger weight was given to sites that are known to be polluted and require remediation because such sites are expected to have a bigger impact than non-polluted sites.

In detail, within each polygon, random points were generated proportional to the polygon's area, with a maximum of 200 points per polygon. These points were assigned weights corresponding to the pollution status of the polygons.

Points that were too close to each other (within 5 meters) were removed to prevent clustering artifacts in the random generation of points.

To ensure the point distribution was representative, the correlation between the area of the polygons and the number of points within them was computed. The final correlation between the number of points in the area and the area was 0.95 and therefore the weight associated with the area is good because the number of points inside each polygon closely matches the polygon area.

*Scatter plot of polygon area vs number of points to generate the heatmap of polluted sites.*

##### **2.3.4 Assessment of years of potential life lost or gained**

Ladoy et al. (7) previously examined how years of life lost or gained (YPLLG) varied across Geneva, using local Moran’s I (LISA) to detect patterns of life expectancy. They looked at ten random subsets of the initial data, each with 10000 individuals, and controlled for factors like sex, nationality, and median age. By doing so, they saw a noticeable decrease in both low and high YPLLG clusters, suggesting that these demographic adjustments played an important role. The YPLLG dataset used in this study was derived from the same mortality database and is independent from the Bus Santé cohort used for AAA1 analyses. No individual-level linkage was performed. Area-level YPLLG values were spatially merged with AAAA1 cluster locations to enable ecological comparisons.

Following the approach described by Ladoy et al., YPLLG was analyzed both unadjusted and adjusted for median age,median income, and nationality, using median (quantile) regression, which is robust to skewed distributions (8). After these demographic adjustments, we went one step further and added environmental factors—specifically NO₂ levels and the density of polluted sites. This was done to understand how these elements interacted with sociodemographic variables to influence YPLLG in AAA1 clusters.

The YPLLG dataset included 21 570 area-level records, with a median age of 40.6 years [IQR 38.3–44.1] and 79.6 % Swiss nationals (95 % CI 79.0–80.1). The median household income of the corresponding residential areas was 114 923 CHF [99 737–147 888], with median values of 24.2 µg/m³ [22.2–26.7] for NO₂ and 46.8 [14.5–110.3] (arbitrary units) for weighted polluted-site density.

Since the AAA1 and YPLLG data came from different sources, it was needed to merge them spatially. To achieve this, circular buffers around each AAA1 point in increments of 50m, 100m, 150m, and 200m were created. For each buffer size, we calculated the median YPLLG and assigned that value to the corresponding AAA1 location.

To identify and retain the most consistently significant YPLLG clusters across random runs, a composite scoring method was applied. This score quantified the frequency and significance of each data point using the formula:

- Composite score = percentage of significant Occurrences × log(occurrence count + 1)

Data points with a composite score above 110 were retained, ensuring robustness across multiple runs. For example, a score of 110 could be achieved if a data point was significant in all (100%) of three runs or in 4 out of 7 runs. The histogram of composite scores is shown below.

Histogram of the composite score.

##### **2.4 Sensitivity analyses and contextual environmental data**

Air pollutants and polluted sites generally correspond to highly industrialised and/or urbanized areas, which are also characterised by water pollution, increased ambient noise, and higher temperatures (9). We therefore performed sensitivity analyses to explore the robustness of the associations between AAA1 and environmental exposures beyond air and soil pollution. These analyses included drinking water supply, night-time road and railway noise, and land surface temperature, as well as a sensitivity analysis including previously excluded participants to confirm that their exclusion did not affect the results (see Supplementary Results 3.1).

##### **2.4.1 Drink water data collection**

Drinking water supply zones were mapped using official data from Geneva’s public utility services. Each residential location was assigned to a drinking water distribution zone based on its geographic position. Water quality parameters, including nitrate concentration, hardness, calcium, and magnesium levels, were obtained at the zone level and used as contextual environmental indicators. These data were considered in sensitivity analyses to assess whether AAA1 spatial patterns overlapped with areas supplied by water sources known to be affected by chronic pollution.

##### **2.4.2 Noise pollution data collection**

Information on noise pollution during 2015 came from the sonBASE database, a detailed, science-based resource maintained by the Federal Office for the Environment (FOEN). Available since 2009, this database consolidates inputs from multiple federal agencies and geospatial frameworks, adhering to both the Noise Abatement Ordinance and EU environmental noise directives. By integrating traffic counts and modelling results, it covers over 68000 kilometres of roads and 3000 kilometres of railway lines. This extensive coverage makes it possible to evaluate noise levels, particularly from road and rail traffic, across all of Switzerland with a high degree of accuracy (10).

##### **2.4.3 Land surface temperature data collection**

To examine land surface temperatures (LST), cloud-free images from the Landsat 8 satellite were processed using Google Earth Engine (GEE), following the guidelines outlined by (11). The focus was on the summer months of 2015 (June through August) and the resolution of Landsat 8 images was 30m.

##### **2.5 SCORE2 computation**

SCORE2 and SCORE2-OP (hereinafter, SCORE2) risk scores, predicting 10-year risk of fatal and non-fatal atherosclerotic cardiovascular disease in participants aged 40-69 years and 70-89 years, respectively, were computed based on traditional CV risk factors including age, sex, systolic blood pressure, smoking status, diabetes, and total and HDL cholesterol levels (12). SCORE2 has been externally validated for use in the Swiss population (13). SCORE2 were used to adjust AAA1 clusters for traditional CV risk factor in a single continuous variable, but it was not applied to YPLLG-related analyses for the reason explained in paragraph 2.3.3.

##### **2.6 Statistics**

In the geographically weighted regression (GWR) stage, it was essential to ensure a robust local context. To achieve this, points surrounded by fewer than 15 neighbours within a 900-meter radius were omitted. Removing these isolated data points reduced the dataset to 1602 observations, concentrating the analysis on areas rich in contextual data, such as AAA1 hotspots and the city centre of Geneva.

GWR was then performed with a 1000-meter bandwidth, consistent with the scale used in previous LISA analyses. Implemented via Python’s mgwr package (14) GWR captured how relationships between AAA1 levels, environmental pollutants, and YPLLG changed from one location to another. For the GWR analysis, a bi-squared kernel was used to see if we could find very local associations. The method yielded localized t-values, allowing for the identification of specific neighbourhoods where pollutant impacts reached statistical significance (p < 0.05).

2.7 Adjustments

Direct acyclic graph (DAG):

**(Left)** Pollution → AAA1 (*Bus Santé*): Pollution is hypothesized to influence AAA1. Individual cardiovascular risk factors (age, sex, blood pressure, cholesterol, smoking) were accounted for by regressing AAA1 on SCORE2. Income, shown conceptually, may affect both pollution and AAA1; genetics and infections are unmeasured determinants.
**(Right)** AAA1 → YPLLG (ecological overlay): Pollution and area-level covariates (income, age structure, nationality) act as common causes of both AAA1 and YPLLG and were included in the YPLLG adjustment. The overlay combines independent datasets (*Bus Santé* for AAA1; mortality registry for YPLLG) and is cross-sectional, causality is not implied.

**3. Results**

##### **3.1 Sensitivity analysis of excluded participants**

Among the participants included in the analysis, the median age was 45.6 years (IQR: 35.8-56.5), and the median AAA1 level was 0.46 (IQR: 0.31-0.65). Among those excluded, the median age was 47.3 years (IQR: 36.0-59.4), and the median AAA1 level was 0.43 (IQR: 0.28-0.60). No significant differences were found in mean age (p = 0.53) or AAA1 levels (p = 0.1) between the individuals that were excluded from the analysis and those that were kept.

Analyses repeated on the full dataset (N = 1987) yielded clustering and YPLLG results consistent with those of the main analysis, confirming that the exclusions did not affect the findings.

***Supplementary Figure 1****: Comparison of bandwidths using LISA (Local Indicators of Spatial Association). Panels a–d represent spatial lags of 800m, 1000m, 1200m, and 1400m, respectively. The figure displays Local Moran’s I values for AAA1 levels (2016–2018, ages 20–75) in the city of Geneva. Significance was assessed at p < 0.05 using 999 permutations. Red buffers represent high-high clusters (high values surrounded by high values), blue buffers represent low-low clusters (low values surrounded by low values), teal dots indicate low-high clusters (low values surrounded by high values), and pink dots indicate high-low clusters (high values surrounded by low values). The different bandwidths show consistent clustering patterns. For example, the high-high clusters in the Jonction area stayed consistent with all bandwidths and with the 1400m bandwidth, the AAA1 high-high clusters covered a slightly broader region.*

***Supplementary Figure 2****: Local Moran’s I of adjusted AAA1 (2016-2018, ages 20-75) for the city of Geneva, overlaid with sources and geographic water distribution. The dataset contains n = 1867 observations. The spatial distance was set to 1000 meters, with a significance level of 0.05 and 999 permutations. Red buffers indicate high-high clusters (e.g., high values surrounded by high mean values, n=93), blue buffers indicate low-low clusters (low values surrounded by low mean values, n=51), teal dots indicate low-high clusters (low values surrounded by high mean values, n=103), and pink dots indicate high-low clusters (high values surrounded by low mean values, n=40). The high-high clusters are present in the city centre and in Region 4 (highlighted in orange colour) which is known to be polluted by the Arve river. See the main paper for further details.*

***Supplementary Figure 3*** *: AAA1 hotspots in Geneva are shown in red, overlaid with PM_2.5_ levels categorized into three equal quantile classes: first quantile (yellow), second quantile (purple), and third quantile (dark). The overlap of high AAA1 concentrations (hotspots) with areas of higher PM_2.5_ concentrations suggests a possible link between exposure to fine particulate matter and elevated AAA1 levels.*

***Supplementary Figure 4****: AAA1 hotspots in Geneva are shown in red, overlaid with PM_10_ levels categorized into three equal quantile classes: first quantile (yellow), second quantile (pink), and third quantile (dark). Similar observations are found between PM_10_ and PM_2.5_.*

| **Measure (Meteotest)** | **NO₂ (µg/L)** | **PM₁₀ (µg/L)** | **PM₂.₅ (µg/L)** |
| --- | --- | --- | --- |
| Pollutant levels in high-high AAA1 | 26.90  (24.40 ; 30.20) | 17.20  (16.40 ; 18.00) | 11.60  (11.20 ; 12.10) |
| Pollutant Levels in low-low AAA1 | 25.70  (22.20 ; 26.50) | 16.70  (15.95 ; 17.00) | 11.20  (10.90 ; 11.50) |
| Pollutant levels in high-low AAA1 | 24.55  (21.12 ; 25.62) | 16.30  (15.30 ; 16.90) | 11.05  (10.50 ; 11.40) |
| Pollutant levels in low-high AAA1 | 27.10  (24.40 ; 30.55) | 17.20  (16.40 ; 18.05) | 11.60  (11.30 ; 12.10) |
| Pollutant levels in NSD AAA1 | 23.70  (21.40 ; 26.21) | 16.30  (15.50 ; 17.00) | 11.10  (10.60 ; 11.50) |
| Kruskal-Wallis p-value | <0.001 | <0.001 | <0.001 |
| Mann-Whitney p; high-high vs low-low | <0.001 | <0.001 | <0.001 |
| Mann-Whitney p; high-high vs NSD | <0.001 | <0.001 | <0.001 |
| Mann-Whitney p-value: high-high vs high-low | <0.001 | <0.001 | <0.001 |
| Mann-Whitney p-value: high-high vs low-high | 0.675 | 0.690 | 0.555 |
| Mann-Whitney p; NSD vs low-low | 0.109 | 0.223 | 0.413 |

***Supplementary Table 1****: Statistical summary for NO₂, PM₁₀, and PM₂.₅ levels across different AAA1 cluster types, including high-high, low-low, high-low, and low-high groups. Results are expressed as median (IQR). We can see that in the high-high AAA1 clusters, pollutant levels are higher than in the neutral areas (NSD) (p<0.001). However, we can see that the discordant cluster (low-high clusters) have similar pollution level than high-high clusters. NSD stands for no spatial dependence and corresponds to the neutral areas.*

| Measure | Density without weight  Median (IQR) | Density with weight  Median (IQR) |
| --- | --- | --- |
| Polluted sites in high-high AAA1 | 55.45 (13.57 ; 143.55) | 66.75 (13.74 ; 273.63) |
| Polluted sites in low-low AAA1 | 44.93 (20.90 ; 79.77) | 50.37 (23.58 ; 83.41) |
| Polluted sites in high-low AAA1 | 33.62 (8.25 ; 80.52) | 36.12 (8.25 ; 83.35) |
| Polluted sites in low-high AAA1 | 66.48 (18.91 ; 128.13) | 73.53 (18.91 ; 249.51) |
| Polluted sites in NSD class | 48.10 (11.31 ; 99.29) | 52.07 (11.51 ; 113.18) |
| Kruskal-Wallis p-value | 0.067 | 0.004 |
| Mann-Whitney p-value; high-high vs low-low | 0.467 | 0.267 |
| Mann-Whitney p-value; high-high vs NSD | 0.075 | 0.008 |
| Mann-Whitney p-value: high-high vs high-low | 0.355 | 0.169 |
| Mann-Whitney p-value: high-high vs low-high | 0.789 | 0.952 |
| Mann-Whitney p-value; NSD vs low-low | 0.859 | 0.977 |

***Supplementary Table 2****: Statistical summary for the impact of polluted sites, including both density without weighting and weighted polluted sites density, across different AAA1 clusters. The weighted polluted sites density reveals a significant difference between the high-high AAA1 clusters and the no spatial dependence class (NSD, p = 0.008). However, differences in polluted sites between high-high and low-low clusters are not statistically significant.*

**3.2.4 Association between AAA1 and years of life lost or gained**

Supplementary Table 3 provides a summary of the median YPLLG values across different buffer sizes (50, 100, 150, and 200 meters) for high-high (HH), low-low (LL), high-low (HL), low-high (LH), and no spatial dependence (NSD) classes. Supplementary Table 4 presents the statistical significance of the differences in YPLLG values between AAA1 clusters across various buffer distances.

The non-adjusted and adjusted analyses indicate that, across all buffer sizes, the HH and LH AAA1 clusters are associated with significantly lower median YPLLG values than the LL and neutral class (NSD). The effects remain significant after adjustments for age, income, nationality, NO₂ exposure, and proximity to polluted sites.

At the 50-meter buffer, the unadjusted median YPLLG in HH clusters was 2.69 years, compared to 4.41 years in LL clusters and 9.49 years in NSD class. The difference between HH and NSD class was -6.80 years (p < 0.001). After adjustment for median age, income, and nationality, the median YPLLG in HH clusters decreased to -5.17 years, while in NSD class it was 0.19 years, resulting in a difference of -5.36 years (p < 0.001). After full adjustment for age, income, nationality, NO₂ levels and proximity to polluted sites, the median YPLLG in HH clusters was -4.30 years, compared to 0.13 years in NSD class, yielding a difference of -4.43 years (p < 0.001). The YPLLG values between HH and LL AAA1 clusters at this buffer size were not statistically significant (p > 0.05).

At the 100 m buffer, the discrepancies between HH clusters and both LL and NSD classes became more evident and statistically significant. The fully adjusted median YPLLG in HH AAA1 clusters was -3.13 years, in comparison to 0.78 years in LL clusters and 0.24 years in NSD class. The difference between HH and LL clusters was -3.91 years (p < 0.001), and between HH and NSD classes was -3.37 years (p < 0.001).

As the buffer size increased to 150 m, the differences between HH clusters and both LL and NSD classes remained statistically significant, although the magnitude of the differences decreased. The fully adjusted median YPLLG in HH clusters was found to be -1.52 years, in comparison to 0.76 years in LL clusters and 0.23 years in NSD class. The difference between HH and LL clusters was -2.28 years (p = 0.007), and between HH and NSD class was -1.75 years (p < 0.001).

At the 200 m buffer, the fully adjusted median YPLLG in HH clusters was -0.75 years, while in LL clusters it was 3.60 years and in NSD class 0.24 years. The difference between HH and LL clusters was -4.35 years (p < 0.001), and between HH and NSD classes was -0.99 years (p = 0.005).

Despite a reduction in effect size as the buffer size increased, AAA1 HH clusters consistently had lower levels of YPLLG, (i.e. they lost more years of life) when compared to LL or NSD AAA1 classes. This association remained robust after adjustment and highlights the potential public health impact of AAA1 exposure. These findings indicate that high AAA1 levels are associated with significant reductions in life expectancy, particularly when compared to areas with low AAA1 levels or neutral areas.

| Buffer Distance (m) | Model | AAA1 HH [N] | AAA1 LL [N] | AAA1 NSD [N] | AAA1 HL [N] | AAA1 LH [N] |
| --- | --- | --- | --- | --- | --- | --- |
| 50m | 1 | 2.69 (-14.63 ; 13.20) [481] | 4.41 (-16.09 ; 13.88) [195] | 9.49 (-4.08 ; 19.07) [21485] | 9.02 (-8.36 ; 19.61) [228] | 3.07 (-12.47 ; 15.03) [612] |
|  | 2 | -5.17 (-20.45 ; 5.71) [481] | -2.72 (-23.07 ; 7.55) [195] | 0.19 (-12.83 ; 9.82) [21485] | 2.41 (-14.42 ; 12.20) [228] | -4.15 (-20.22 ; 6.42) [612] |
|  | 3 | -4.30 (-20.08 ; 6.86) [481] | -3.40 (-22.80 ; 6.80) [195] | 0.13 (-12.75 ; 9.68) [21485] | 2.08 (-14.70 ; 11.89) [228] | -3.13 (-18.67 ; 7.74) [612] |
| 100m | 1 | 4.41 (-13.33 ; 14.61) [1159] | 7.27 (-10.08 ; 18.61) [515] | 9.77 (-3.72 ; 19.29) [21485] | 9.07 (-7.06 ; 19.63) [582] | 4.00 (-11.60 ; 14.94) [1493] |
|  | 2 | -4.30 (-21.17 ; 6.81) [1159] | 0.23 (-17.68 ; 11.69) [515] | 0.33 (-12.48 ; 9.88) [20098] | 2.39 (-13.54 ; 12.60) [582] | -3.55 (-19.45 ; 6.79) [1493] |
|  | 3 | -3.13 (-20.28 ; 7.83) [1159] | 0.78 (-17.47 ; 12.64) [515] | 0.24 (-12.39 ; 9.68) [20098] | 2.56 (-14.07 ; 12.94) [582] | -2.70 (-18.05 ; 7.99) [1493] |
| 150m | 1 | 5.60 (-11.17 ; 16.08) [1883] | 7.91 (-9.37 ; 18.42) [732] | 9.77 (-3.40 ; 19.35) [19138] | 8.07 (-8.97 ; 19.00) [751] | 5.27 (-10.98 ; 15.64) [2364] |
|  | 2 | -2.70 (-18.80 ; 8.20) [1883] | 0.39 (-16.20 ; 11.46) [732] | 0.33 (-12.32 ; 9.84) [19138] | 0.73 (-16.80 ; 11.68) [751] | -3.06 (-18.88 ; 7.34) [2364] |
|  | 3 | -1.52 (-17.74 ; 9.37) [1883] | 0.76 (-16.59 ; 12.09) [732] | 0.23 (-12.27 ; 9.61) [19138] | 0.89 (-16.71 ; 12.10) [751] | -2.47 (-17.75 ; 8.21) [2364] |
| 200m | 1 | 7.20 (-9.08 ; 17.35) [2465] | 12.08 (-0.98 ; 21.88) [1254] | 9.67 (-3.72 ; 19.18) [18083] | 6.96 (-9.78 ; 18.35) [903] | 5.96 (-10.47 ; 16.32) [3055] |
|  | 2 | -1.66 (-17.27 ; 9.06) [2465.0] | 3.48 (-9.07 ; 12.91) [1254] | 0.85 (-11.15 ; 10.85) | -0.04 (-17.79 ; 11.27) [903] | -2.36 (-17.96 ; 7.86) [3055] |
|  | 3 | -0.75 (-16.12 ; 9.83) [2465] | 3.60 (-8.76 ; 12.74) [1254] | 0.24 (-12.47 ; 9.72) [18083] | 0.08 (-18.00 ; 11.75) [903] | -1.71 (-17.18 ; 8.62) [3055] |

***Supplementary Table 3:*** *Median (IQR) YPLLG values for different buffer sizes around AAA1 clusters, including high-high, low-low, high-low, low-high, and neutral (NSD) classes. Results are presented for both non-adjusted and adjusted models. Model 1, unadjusted. Model 2, adjusted for median age, income, and nationality. Model 3, adjusted for median age, income, nationality, NO₂ levels, and polluted sites where applicable.*

| Buffer Distance (m) | Model | HH vs. LL p-value | HH vs. NSD p-value | HH vs. HL p-value | HH vs. LH p-value | LL vs. NSD p-value |
| --- | --- | --- | --- | --- | --- | --- |
| 50m | 1 | 0.783 | <0.001 | <0.001 | 0.317 | <0.001 |
|  | 2 | 0.375 | <0.001 | <0.001 | 0.366 | 0.005 |
|  | 3 | 0.861 | <0.001 | <0.001 | 0.489 | 0.002 |
| 100m | 1 | 0.002 | <0.001 | <0.001 | 0.558 | 0.003 |
|  | 2 | <0.001 | <0.001 | <0.001 | 0.479 | 0.910 |
|  | 3 | <0.001 | <0.001 | <0.001 | 0.440 | 0.417 |
| 150m | 1 | 0.015 | <0.001 | 0.008 | 0.610 | <0.001 |
|  | 2 | <0.001 | <0.001 | <0.001 | 0.360 | 0.790 |
|  | 3 | 0.007 | <0.001 | 0.003 | 0.142 | 0.413 |
| 200m | 1 | <0.001 | <0.001 | <761 | 0.042 | <0.001 |
|  | 2 | <0.001 | <0.001 | 0.021 | 0.113 | <0.001 |
|  | 3 | <0.001 | 0.005 | 0.150 | 0.062 | <0.001 |

***Supplementary Table 4****: Mann-Whitney U Test results for YPLLG values within different buffer sizes (50m, 100m, 150m, and 200m) across AAA1 clusters (high-high, low-low, high-low, low-high, and no spatial dependence (NSD)). Results are presented for both non-adjusted and adjusted models. Model 1, unadjusted. Model 2, adjusted for median age, income, and nationality. Model 3, adjusted for median age, income, nationality, NO₂ levels, and polluted sites where applicable.*

Supplementary Table 5 provides a detailed statistical summary of these environmental variables within the YPLLG clusters, including median values, interquartile ranges (IQR), and results from the Kruskal-Wallis and Mann-Whitney U tests.

The data indicate significant differences in environmental pollutant levels among the clusters. High-high YPLLG clusters are associated with lower median levels of NO₂, PM₁₀, and PM₂.₅ compared to low-low clusters and others. For instance, the median NO₂ level (Meteotest data) in high-high clusters is 22.95 µg/m³, whereas it is 27.45 µg/m³ in low-low clusters and 24.20 µg/m³ in others, with a Kruskal-Wallis p-value of <0.001. Similar patterns are observed for PM₁₀ and PM₂.₅ levels. Additionally, high-high clusters exhibit lower night traffic noise levels and lower land surface temperatures (LST) compared to low-low clusters, suggesting a correlation between environmental quality and increased life expectancy.

These findings imply that regions with higher YPLLG are characterized by better environmental conditions, supporting the hypothesis that reduced exposure to pollutants is associated with improved health outcomes.

|  |  |  |  |  | **Mann-Whitney p-value** | | |
| --- | --- | --- | --- | --- | --- | --- | --- |
| Measure | **High [2,732]** | **Low [1,368]** | **Others [18,651]** | **Kruskal- Wallis test p-value** | **HH vs. LL** | **HH vs. Others** | **LL vs. Others** |
| NO_2_ levels (µg/L) [Meteotest] | 22.95  (21.60 ; 24.80) | 27.45  (26.60 ; 29.20) | 24.20  (22.10 ; 26.60) | <0.001 | <0.001 | <0.001 | <0.001 |
| PM_10_ levels (µg/L) [Meteotest] | 15.95  (15.70 ; 16.20) | 17.70  (17.30 ; 18.10) | 16.35  (15.70 ; 17.00) | <0.001 | <0.001 | <0.001 | <0.001 |
| PM_2.5_ levels (µg/L) [Meteotest] | 10.90  (10.80 ; 11.10) | 11.90  (11.70 ; 12.10) | 11.10  (10.70 ; 11.50) | <0.001 | <0.001 | <0.001 | <0.001 |
| NO_2_ levels (µg/L) [G2AME] | 26.27  (24.89 ; 31.32) | 33.33  (32.42 ; 33.77) | 30.27  (25.46 ; 32.59) | <0.001 | <0.001 | <0.001 | <0.001 |
| PM_10_ levels (µg/L) [G2AME] | 22.87  (22.19 ; 23.28) | 24.38  (24.12 ; 24.65) | 23.28  (22.44 ; 23.96) | <0.001 | <0.001 | <0.001 | <0.001 |
| Night traffic noise | 42.00  (38.00 ; 49.50) | 47.00  (43.00 ; 52.00) | 44.00  (39.00 ; 49.50) | <0.001 | <0.001 | 0.014 | <0.001 |
| Night train noise | 0.00  (0.00 ; 0.00) | 0.00  (0.00 ; 10.00) | 0.00  (0.00 ; 12.00) | <0.001 | <0.001 | <0.001 | 0.061 |
| Land surface temperature | 23.61  (22.89 ; 24.57) | 25.13  (23.98 ; 25.88) | 23.86  (22.73 ; 25.03) | <0.001 | <0.001 | <0.001 | <0.001 |
| Polluted sites | 10.63  (3.21 ; 37.55) | 117.57  (49.63 ; 181.78) | 45.81  (14.98 ; 105.82) | <0.001 | <0.001 | <0.001 | <0.001 |
| Polluted sites with weight | 10.63  (3.21 ; 37.55) | 147.90  (56.70 ; 288.28) | 49.60  (15.69 ; 110.58) | <0.001 | <0.001 | <0.001 | <0.001 |

***Supplementary Table 5****: Statistical summary for environmental variables within YPLLG clusters, including high-high, low-low, and other categories. Median (IQR) values are provided for each variable, along with Kruskal-Wallis and Mann-Whitney U test p-values for comparisons between the clusters.*

Supplementary Table 6 presents Pearson correlation coefficients between YPLLG and various environmental pollution indicators. The results show negative correlations between YPLLG and pollutants such as NO₂, PM₁₀, PM₂.₅, night train noise, and land surface temperature. For example, the correlation coefficient for NO₂ (Meteotest) is -0.076 (p < 0.001), indicating that higher NO₂ levels are associated with lower YPLLG.

| **Column** | **Pearson** | **P-value (Pearson)** | **Spearman** | **P-value (Spearman)** |
| --- | --- | --- | --- | --- |
| NO_2_ (Meteotest) | -0.076 | <0.001 | -0.071 | <0.001 |
| PM_10_ (Meteotest) | -0.082 | <0.001 | -0.082 | <0.001 |
| PM_2.5_ (Meteotest) | -0.083 | <0.001 | -0.081 | <0.001 |
| Night traffic noise | -0.005 | 0.469 | -0.011 | 0.102 |
| Night train noise | -0.024 | <0.001 | -0.036 | <0.001 |
| Land surface temperature | -0.063 | <0.001 | -0.068 | <0.001 |
| Polluted sites | -0.075 | <0.001 | -0.103 | <0.001 |
| Polluted sites with weight | -0.099 | <0.001 | -0.115 | <0.001 |

***Supplementary Table 6****: Pearson and Spearman correlation coefficients between YPLLG and environmental pollution indicators, including NO₂, PM_10_, PM_2.5_, night traffic noise, night train noise, land surface temperature, and polluted sites (with and without weighting).*

Supplementary Figure 10a-h displays the results of geographically weighted regression (GWR) analyses, showing the spatial distribution of t-values for the associations between AAA1 and environmental pollutants, as well as between AAA1 and YPLLG, across the study area. The AAA1 high-high clusters are highlighted in red, and the classification of t-values follows the Student’s t distribution. Supplementary Table 7 summarizes these GWR results, including the number of significant positive and negative t-values and the global beta coefficients for each variable.

***Supplementary Figure 5a-b****: GWR results showing the spatial distribution of t-values across the study area. Panel a displays the results for NO₂ and AAA1, while panel b shows the results for PM_10_ and AAA1. The significance of the t-values is based on Student's t-distribution, with thresholds set at p < 0.01 and p < 0.05. AAA1 high-high clusters are presented in red.*

***Supplementary Figure 5c-d****: GWR results showing the spatial distribution of t-values across the study area. Panel c displays the results for PM_2.5_ and AAA1, while panel d shows the results for polluted sites and AAA1.* *The significance of the t-values is based on Student's t-distribution, with thresholds set at p < 0.01 and p < 0.05. AAA1 high-high clusters are presented in red.*

***Supplementary Figure 5e-f****: GWR results showing the spatial distribution of t-values across the study area. Panel e displays the results for weighted polluted sites and AAA1, while panel f shows the results for night traffic noise and AAA1. The significance of the t-values is based on Student's t-distribution, with thresholds set at p < 0.01 and p < 0.05. AAA1 high-high clusters are presented in red.*

***Supplementary Figure 5g-h****: GWR results showing the spatial distribution of t-values across the study area. Panel g displays the results for land surface temperature and AAA1, while panel h shows the results for years of life lost or gained (YPLLG) and AAA1. The significance of the t-values is based on Student's t-distribution, with thresholds set at p < 0.01 and p < 0.05. AAA1 high-high clusters are presented in red.*

| **Variable** | **Total N** | **Non-significant t-values** | **Positive t-values (*p*<0.05)** | **Negative t-values (*p*<0.05)** | **Global beta (p-value)** |
| --- | --- | --- | --- | --- | --- |
| NO_2_ | 1602 | 1560 | 36 | 6 | 0.054 (0.033) |
| PM_10_ | 1602 | 1388 | 27 | 0 | 0.053 (0.036) |
| PM_2.5_ | 1602 | 1374 | 47 | 0 | 0.059 (0.020) |
| Polluted sites | 1602 | 1315 | 100 | 11 | 0.021 (0.404) |
| Weighted polluted sites | 1602 | 1332 | 89 | 0 | 0.034 (0.184) |
| Night traffic noise | 1602 | 1369 | 60 | 1 | 0.043 (0.089) |
| LST | 1602 | 1255 | 53 | 118 | 0.002 (0.952) |
| YPLLG | 1569 | 1320 | 25 | 79 | 0.006 (0.825) |

***Supplementary Table 7****: Summary of GWR results, including the total number of observations (N), non-significant t-values, positive t-values (p < 0.05), negative t-values (p < 0.05), and global beta values with corresponding p-values for NO₂, PM_10_, PM_2.5_, polluted sites, weighted polluted sites, night traffic noise, land surface temperature (LST), and YPLLG. Note: While the global regression model showed minimal explanatory power for most variables, the GWR analysis provided improved explanatory power. For instance, the global regression model yielded an R² of approximately 0.003 for the relationship modelled as AAA1 ~ NO₂, whereas the GWR model achieved a higher R² of 0.073.*

| **Parameter** | **Region 1** | **Region 2** | **Region 3** | **Region 4** |
| --- | --- | --- | --- | --- |
| Nitrates (mg/L) | 2.4  (1.8 ; 2.8) | 2.8  (1.5 ; 3.0) | 2.4  (1.6 ; 2.9) | 4.4  (2.4 ; 5.8) |
| Water Hardness (°F) | 13.4  (12.3 ; 14.3) | 13.4  (12.6 ; 14.4) | 13.5  (12.9 ; 14.1) | 19.6  (13.6 ; 21.8) |
| Calcium (mg/L) | 43.7  (40.3 ; 46.6) | 43.8  (41.3 ; 47.1) | 43.9  (42.0 ; 45.7) | 62.4  (44.3 ; 68.5) |
| Magnesium (mg/L) | 6.0  (5.6 ; 6.5) | 6.0  (5.5 ; 6.4) | 6.0  (5.8 ; 6.4) | 9.7  (6.1 ; 12.8) |

***Supplementary Table 8****: Water quality parameters across different zones in Geneva. The results are presented as mean (min ; max).*

As shown in Supplementary Table 9, the high-high AAA1 clusters have a median night traffic noise level of 47.00 dB, significantly higher than the 43.00 dB observed in the neutral class (no spatial dependence, p < 0.001). However, high-high AAA1 clusters don’t have higher temperatures than other clusters (Supplementary Table 10).

| **Measure** | **Night traffic (dB)** | **Night train (dB)** |
| --- | --- | --- |
| Pollutant levels in high-high AAA1 | 47.0 (42.0 ; 55.0) | 4.0 (0.0 ; 15.0) |
| Pollutant levels in low-low AAA1 | 45.0 (40.0 ; 48.0) | 22.0 (0.5 ; 27.5) |
| Pollutant levels in high-low AAA1 | 44.0 (40.0 ; 46.3) | 2.0 (0.0 ; 24.5) |
| Pollutant levels in low-high AAA1 | 48.0 (44.0 ; 56.0) | 2.0 (0.0 ; 12.5) |
| Pollutant levels in NSD AAA1 | 43.0 (37.0 ; 48.0) | 0.0 (0.0 ; 8.0) |
| Kruskal-Wallis p-value | <0.001 | <0.001 |
| Mann-Whitney p-value: high-high vs low-low | 0.001 | <0.001 |
| Mann-Whitney p-value: high-high vs NSD | <0.001 | <0.001 |
| Mann-Whitney p-value: high-high vs high-low | 0.011 | 0.343 |
| Mann-Whitney p-value: high-high vs low-high | 0.403 | 0.489 |
| Mann-Whitney p-value: NSD vs low-low | 0.088 | <0.001 |

***Supplementary Table 9****: Statistical summary (median and IQR) for night traffic and train noise levels across different AAA1 clusters, including Mann-Whitney U and Kruskal-Wallis p-values.*

| **Measure** | **LST (°C)** |
| --- | --- |
| LST High-High AAA1 | 24.11 (22.52 ; 25.29) |
| LST Low-Low AAA1 | 25.07 (24.21 ; 26.18) |
| LST High-Low AAA1 | 25.04 (23.73 ; 26.11) |
| LST Low-High AAA1 | 24.41 (23.01 ; 25.55) |
| LST in NSD AAA1 | 24.00 (22.83 ; 25.12) |
| Kruskal-Wallis p-value | <0.001 |
| Mann-Whitney p-value: high-high vs low-low | 0.001 |
| Mann-Whitney p-value: high-high vs NSD | 0.622 |
| Mann-Whitney p-value: high-high vs high-low | 0.023 |
| Mann-Whitney p-value: high-high vs low-high | 0.259 |
| Mann-Whitney p-value: NSD vs low-low | <0.001 |

***Supplementary Table 10****: Statistical summary (median and IQR) for land surface temperature (LST) across different AAA1 clusters, including Mann-Whitney U and Kruskal-Wallis p-values.*

*References*

1.	Hafiane A, Favari E, Daskalopoulou SS, Vuilleumier N, Frias MA. High-density lipoprotein cholesterol efflux capacity and cardiovascular risk in autoimmune and non-autoimmune diseases. Metabolism. 2020 Mar 1;104:154141.

2.	Heldstab J, Schäppi B, Künzle T. Aktualisierung des PolluMap- Modells für 2015, 2020 und 2030. Berne: Bundesamt für Umwelt (BAFU); 2020.

3.	Office fédéral de l’environnement (OFEV / BAFU). La qualité de l’air en 2018. 2018; Available from: https://www.bafu.admin.ch/bafu/fr/home/themes/air/publications-etudes/publications/la-qualite-de-l-air-en-2018.html

4.	SITG – République et Canton de Genève. Catalogue des données géographiques [Internet]. [cited 2024 July 17]. Available from: https://ge.ch/sitg/sitg_catalog/sitg_donnees?keyword=&geodataid=3500&topic=tous&service=tous&datatype=tous&distribution=tous&sort=auto#

5.	Netek R, Pour T, Slezakova R. Implementation of Heat Maps in Geographical Information System – Exploratory Study on Traffic Accident Data. Open Geosciences. 2018 Jan 1;10(1):367–84.

6.	Huang G, Wang X, Chen D, Wang Y, Zhu S, Zhang T, et al. A hybrid data-driven framework for diagnosing contributing factors for soil heavy metal contaminations using machine learning and spatial clustering analysis. J Hazard Mater. 2022 Sept 5;437:129324.

7.	Ladoy A, Vallarta-Robledo JR, De Ridder D, Sandoval JL, Stringhini S, Da Costa H, et al. Geographic footprints of life expectancy inequalities in the state of Geneva, Switzerland. Sci Rep. 2021 Dec 2;11:23326.

8.	McGreevy KM, Lipsitz SR, Linder JA, Rimm E, Hoel DG. Using median regression to obtain adjusted estimates of central tendency for skewed laboratory and epidemiologic data. Clin Chem. 2009 Jan;55(1):165–9.

9.	Montes-González D, Vílchez-Gómez R, Barrigón-Morillas JM, Atanasio-Moraga P, Rey-Gozalo G, Trujillo-Carmona J. Noise and Air Pollution Related to Health in Urban Environments. Proceedings. 2018;2(20):1311.

10.	Office fédéral de l’environnement (OFEV). Pollution sonore en Suisse [Internet]. [cited 2024 Oct 8]. Available from: https://www.bafu.admin.ch/bafu/fr/home/themen/thema-laerm/laerm--publikationen/publikationen-laerm/laermbelastung-in-der-schweiz-sonbase.html

11.	Ermida SL, Soares P, Mantas V, Göttsche FM, Trigo IF. Google Earth Engine Open-Source Code for Land Surface Temperature Estimation from the Landsat Series. Remote Sensing. 2020 Jan;12(9):1471.

12.	SCORE2-OP working group and ESC Cardiovascular risk collaboration. SCORE2-OP risk prediction algorithms: estimating incident cardiovascular event risk in older persons in four geographical risk regions. Eur Heart J. 2021 July 1;42(25):2455–67.

13.	Delabays B, de La Harpe R, Vollenweider P, Fournier S, Müller O, Strambo D, et al. Comparison of the European and US guidelines for lipid-lowering therapy in primary prevention of cardiovascular disease. Eur J Prev Cardiol. 2023 Nov 1;30(17):1856–64.

14.	Oshan TM, Li Z, Kang W, Wolf LJ, Fotheringham AS. mgwr: A Python Implementation of Multiscale Geographically Weighted Regression for Investigating Process Spatial Heterogeneity and Scale. ISPRS Int J Geoinf. 2019 June;8(6):269.
